# Editorial: Health promotion in the universities and other educational settings

**DOI:** 10.3389/fpsyg.2025.1662700

**Published:** 2025-08-05

**Authors:** Miguel Corbí, Antoni Aguiló Pons, Carmen Gallardo-Pino, Mario Del Líbano, Carlos Emanuel Rodriguez-Diaz, Hiram Arroyo-Acevedo

**Affiliations:** ^1^Specific Didactics, University of Burgos, Burgos, Spain; ^2^Faculty of Nursing and Physiotherapy, Universitat de les Illes Balears, Palma, Spain; ^3^Department of Medical Specialization and Public Health, Universidad Rey Juan Carlos, Móstoles, Spain; ^4^Faculty of Educational Sciences, University of Burgos, Burgos, Spain; ^5^Department of Community Health Sciences, Boston University, Boston, MA, United States; ^6^Social Sciences, Universidad de Puerto Rico, San Juan, Puerto Rico

**Keywords:** university health promotion, wellbeing in higher education, healthy lifestyles, student mental health, interinstitutional collaboration

Over recent decades, universities have progressively assumed a more active role as health-promoting environments, responding to the pressing challenges that affect student populations in physical, psychological, and social dimensions, and meeting the society demands. Far from being mere centers of academic transmission, universities are increasingly recognized as pivotal agents for fostering healthy citizenship, with the capacity to shape habits, attitudes, and systems of care that resonate beyond the campus walls. As articulated in the foundational reflections of the *Movimiento de Universidades Promotoras de la Salud* (MUPS) and captured in the Ibero-American and Spanish networks (RIUPS, REUPS) (Corbí et al., 2022a), promoting health within universities is both a pedagogical and ethical commitment aligned with sustainability, equity, and community engagement. In this sense, the university becomes not only a setting but a driver of transformation—a laboratory of practices and a platform for intersectoral alliances. Drawing on the salutogenic approach and the model of social health determinants, health promotion in the university context entails a commitment to cultivating empowering environments, participatory policies, and collective capabilities that improve individual and social wellbeing (Corbí et al., 2022b). This vision has been consistently reinforced by initiatives such as active and academic initiatives which reaffirmed the strategic relevance of embedding health in all institutional processes (Corbí Santamaría et al., 2023).

The conceptual foundation underpinning this Research Topic draws on a paradigmatic shift in the field of public health: the transition from a deficit-based model—focused on illness, risk, and medical intervention—toward an asset-based, salutogenic approach that prioritizes empowerment, equity, and the creation of health-enabling environments (Corbí et al., 2022b). This framework promotes the development of individual and collective capabilities through education, participation, and sustainable interventions. In the university setting, this implies reconfiguring campus life, pedagogy, institutional policies, and research to systematically incorporate health as a transversal value. At the forefront of this transformation, the Red Iberoamericana de Universidades Promotoras de la Salud (RIUPS) has played a strategic role, not only in articulating regional action across Latin America and the Iberian Peninsula but also in scaling this model toward a more global reach. Through the dissemination of good practices, collaborative networks, and formative experiences, RIUPS is helping to position universities as key actors in the advancement of the Sustainable Development Goals and in the construction of fairer, healthier societies (Corbí et al., 2022a). This perspective resonates with the ethical imperative of embedding health into the institutional culture of higher education, as expressed in declarations, charters, and inter-university alliances supported by both national and international public health agendas.

The consolidation of health promotion within higher education has been significantly strengthened by institutional collaboration and transnational networks that facilitate the exchange of experiences, knowledge, and strategies. Initiatives such as the *Red Española de Universidades Promotoras de la Salud* (REUPS) and the aforementioned *Red Iberoamericana de Universidades Promotoras de la Salud* (RIUPS) exemplify the power of inter-university cooperation to transcend national boundaries and articulate a shared commitment to public and community health (Corbí et al., 2022a). These networks not only support the dissemination of good practices, but also foster a collective identity based on mutual learning, horizontal governance, and the integration of health into institutional missions. As highlighted in recent contributions, universities across diverse socio-political contexts face common challenges: mental health vulnerabilities, digital dependencies, inequalities in access to healthy environments, and the fragmentation of wellbeing services (Corbí Santamaría et al., 2023). In response, collaboration is not simply desirable—it is imperative. Health promotion cannot remain a peripheral or isolated initiative; it must be embedded into a global academic culture that values equity, participation, sustainability, and social responsibility. The act of sharing—whether practices, policies, or pedagogies—reinforces the idea that promoting healthy habits is not optional, but an institutional obligation across all educational contexts.

In this context, the present Research Topic was conceived as a timely and necessary platform to deepen, systematize, and disseminate current approaches to health promotion within the university setting. The need for such a Research Topic arises from the growing recognition that student populations are experiencing increasingly complex and interrelated health challenges—from psychological distress and digital overexposure to sedentary lifestyles, nutritional imbalances, and academic burnout. These issues, exacerbated by the aftermath of the COVID-19 pandemic, demand urgent and innovative responses from higher education institutions. Beyond the provision of isolated interventions, universities are called to adopt integrated strategies rooted in participation, interdisciplinarity, and evidence-based practice. This Research Topic brings together diverse contributions that reflect this paradigm: empirical studies, institutional programmes, community-based initiatives, and methodological reflections that collectively expand our understanding of what it means to promote health in higher education. By showcasing a broad spectrum of experiences and designs, the Research Topic not only highlights best practices and emerging concerns, but also fosters a critical reflection on the transferability, sustainability, and impact of these actions. Ultimately, the purpose of this Research Topic is to offer a shared space of knowledge construction that strengthens the field of university health promotion and reinforces the academic and civic responsibility of institutions toward the wellbeing of their communities.

The contributions included in this Research Topic reflect the diversity, creativity, and commitment of academics and practitioners engaged in promoting health within universities and other educational settings. A total of 20 articles have been accepted, covering a wide range of health dimensions, methodological approaches, and institutional contexts. These contributions demonstrate how the university can act as a strategic arena for addressing both individual and collective wellbeing through empirical research, evidence-informed interventions, training initiatives, and participatory practices. From psychological wellbeing and emotional resilience to physical activity, digital health literacy, and institutional policy development, the issue captures the multiplicity of pathways through which universities may integrate health into their core functions. To facilitate the interpretation and appreciation of this broad spectrum, the editorial proposes a classification of the contributions into five thematic clusters, each representing a coherent area of focus within the field of health promotion in higher education.

A prominent and recurring axis throughout the accepted contributions concerns the psychological wellbeing and mental health of university students—a theme that has gained increased relevance in the wake of global health crises and ongoing structural pressures in higher education. Several studies examine the psychological and emotional challenges faced by students, including anxiety, depression, emotional dysregulation, academic stress, and perceived loneliness. These are approached through a variety of lenses: the impact of self-compassion and mindfulness-based interventions, the role of social support and stigma in help-seeking behaviors, or the mediating effects of physical activity and mobile phone overuse on depressive symptoms. The articles in this cluster employ both qualitative and quantitative designs, and some are grounded in robust theoretical frameworks such as the broaden-and-build model or coping theories, offering critical insights into the mechanisms and conditions that promote emotional resilience. Collectively, these contributions reflect the urgent need to place mental health at the core of institutional wellbeing agendas and to develop accessible, evidence-informed strategies that support students in their personal, academic, and relational spheres.

Closely aligned with the promotion of psychological wellbeing is a second thematic strand centered on physical activity and the behavioral challenges posed by sedentary lifestyles—issues that are particularly prevalent in university contexts characterized by intense academic demands and prolonged screen exposure. The contributions within this cluster explore how physical activity can be meaningfully integrated into the everyday routines of students and staff, as well as how digital platforms—such as mobile apps, gamified tools, and online interventions—can act as both facilitators and barriers to health engagement. Some articles offer critical insights into the psychological resistances to exercise technologies, while others analyse the motivational and environmental factors that condition physical engagement in campus settings. Underpinning these studies is a shared recognition of the need to reimagine university environments as dynamic and movement-friendly spaces, where physical activity is not an ancillary pursuit but a key component of holistic wellbeing. The articles highlight the potential of blending technological innovation with inclusive health promotion to activate behavioral change, prevent sedentary risk, and reinforce the connections between body, mind, and academic performance.

A third cluster of contributions focuses on structured educational interventions and institutional strategies designed to embed health promotion within the organizational fabric of universities. These studies foreground the transformative role of training and curriculum development in shaping students' health-related perceptions, behaviors, and decision-making processes. Whether through formal modules, workshops, or integrated institutional programmes, the emphasis lies in equipping university populations—not only students but also staff—with the knowledge, skills, and reflexivity needed to engage actively in the co-construction of healthier academic environments. Several contributions evaluate the impact of training on lifestyle practices and the prevention of risky behaviors, while others explore the integration of theoretical models—such as the Job Demands-Resources model or salutogenic principles—into institutional planning. These interventions, often assessed through quasi-experimental or mixed-method designs, reveal the added value of aligning health promotion with the university's educational mission, reinforcing its potential to act as both a learning and living space committed to wellbeing and sustainability.

Another valuable contribution of this Research Topic lies in its attention to the diversity of experiences within university populations, highlighting how health and wellbeing intersect with identity, academic discipline, and social vulnerability. This fourth cluster gathers studies that explore the specific needs, perceptions, and challenges faced by particular groups within the academic community. These include, for example, students in highly demanding fields such as music or medicine, whose physical or emotional wellbeing may be impacted by discipline-specific pressures, performance anxieties, or exposure to risky behaviors. Through qualitative inquiries, survey-based research, and comparative analyses, these contributions shed light on the nuanced ways in which health is shaped by context and lived experience. Importantly, they underscore the necessity of tailoring health promotion strategies to address intersectional realities—considering factors such as gender, professional trajectory, or psychosocial background. By doing so, they help advance a more inclusive and responsive vision of university health, where the complexity of identity is recognized as central to the development of effective and equitable wellbeing initiatives.

The final thematic cluster highlights the methodological and theoretical richness that underpins the current landscape of university health promotion. Several contributions go beyond applied interventions to offer conceptual advancements, critical frameworks, or reflective methodologies that can inform future practice and research. These include qualitative and phenomenological approaches that deepen our understanding of students' lived experiences, as well as theory-driven studies that operationalise models such as psychological reactance theory, broaden-and-build theory, or the Stimulus-Organism-Response paradigm. Such contributions are essential for the consolidation of the field, as they promote analytical clarity, facilitate the replication of effective strategies, and support the development of context-sensitive interventions. By grounding health promotion in rigorous theoretical perspectives and diverse methodological traditions, these works reaffirm the importance of a solid academic foundation for advancing meaningful and sustainable change within university settings.

Taken together, the contributions to this Research Topic reflect not only the diversity and vitality of current efforts in university-based health promotion, but also a shared conviction: that higher education institutions must act with ethical responsibility, scientific rigor, and social commitment in fostering environments that support physical, mental, social, and emotional wellbeing. Across different methodologies, cultural contexts, and disciplinary lenses, these articles converge in their affirmation that promoting health is not a peripheral activity, but a core dimension of the university's mission in the 21st century. As illustrated by the networks and initiatives underpinning this issue—most notably the RIUPS, REUPS initiatives—the collective endeavor to build healthier academic communities must rest on cooperation, critical reflection, and the continual exchange of knowledge. It is hoped that the insights gathered here will serve both as a testament to the work already being done, and as a catalyst for new actions, alliances, and research that continue to position universities as active promoters of health, inclusion, and sustainable development.
